# Adult Onset Immunoglobulin A (IgA) Vasculitis Secondary to Group A Streptococcus Infection

**DOI:** 10.7759/cureus.23987

**Published:** 2022-04-09

**Authors:** Carly E Wallace, Amit Sharma

**Affiliations:** 1 Dermatology, College of Osteopathic Medicine, Lake Erie College of Osteopathic Medicine, Bradenton, USA; 2 Family Medicine, College of Osteopathic Medicine, Lake Erie College of Osteopathic Medicine, Elmira, USA

**Keywords:** immunoglobulin a vasculitis, ig a vasculitis, group a β-hemolytic streptococcus pyogenes, leukocytoclastic vasculitis (lcv), small vessel vasculitis, adult iga vasculitis, henoch-schönlein purpura (iga vasculitis)

## Abstract

Immunoglobulin A (IgA) vasculitis is a small blood vessel vasculitis that is mediated by immune complex deposition. While it is the most common cause of childhood vasculitis, the disease is uncommon in adults with variable clinical manifestations. A 65-year-old female presented with a diffuse erythematous, pruritic, painful rash across her legs, back, and arms of 12 days’ duration. Associated symptoms included fatigue, lower extremity swelling, and migratory arthralgias of the knees and ankles. Skin examination revealed edematous, blanchable, erythematous, annular papules and plaques on the legs, back, and arms with pitting edema of the lower legs. Laboratory testing revealed an elevated erythrocyte sedimentation rate, hypoalbuminemia, proteinuria, hematuria, and a positive antistreptolysin O titer, indicative of recent group A *Streptococcus* infection. Treatment with systemic corticosteroids led to a resolution of all her symptoms. Adult onset IgA vasculitis differs in clinical manifestation and treatment from that of the pediatric population. This case demonstrates the importance of considering IgA vasculitis as a differential diagnosis in adults presenting with small vessel vasculitis.

## Introduction

Immunoglobulin A (IgA) vasculitis, formerly called Henoch-Schönlein purpura, is an immune-mediated small vessel vasculitis associated with IgA deposition, complement deposition, and neutrophil recruitment. IgA vasculitis is the most common systemic vasculitis in children, and in adults, the disease is rare, with an annual incidence of 0.1 to 1.8 per 100,000 individuals [1]. The etiology of IgA vasculitis is unknown; however, it is often post-infectious, with half of all cases being preceded by an upper respiratory infection. Group A *Streptococcus *has been one of the most commonly reported pathogens, with positive throat cultures in 10%-30% of cases and elevated antistreptolysin O (ASO) titers in 20%-50% of cases [2-5]. In addition to environmental factors, several genetic and immune factors have also been implicated in the pathogenesis of IgA vasculitis. Human leukocyte antigen (HLA)-B35 and HLA-DRB1*01 alleles have been associated with susceptibility to IgA vasculitis, along with several other gene polymorphisms [6-8].

According to the European Alliance of Associations for Rheumatology (EULAR), Paediatric Rheumatology International Trials Organisation (PRINTO), and Paediatric Rheumatology European Society (PRES) classification, IgA vasculitis in children is defined as purpura or petechia with lower limb predominance plus one of the four criteria: (1) abdominal pain, (2) arthritis or arthralgia, (3) renal involvement, and (4) leukocytoclastic vasculitis or proliferative glomerulonephritis with predominant IgA deposits [9]. However, there are no set diagnostic criteria for adults with suspected IgA vasculitis, which differs in clinical presentation. In comparison to its presentation in children, adults have a lower frequency of abdominal pain and fever, and a higher frequency of joint symptoms and severe renal involvement. In fact, varying degrees of renal insufficiency have been reported in up to 40% of adults [10,11].

The treatment of IgA vasculitis is mostly supportive, as the disease is typically self-limiting, thus only symptomatic management is necessary. However, corticosteroids are used for widespread and severe cases with colchicine and dapsone reserved for recurrent cases [12]. The management of IgA vasculitis in adults is difficult in that the initial presentation is a poor indicator of the long-term renal outcome, which has not been shown to improve in response to standard therapies [10].

## Case presentation

The patient was a 65-year-old Caucasian female who presented with a diffuse erythematous, pruritic, painful rash across her legs, back, and arms of 12 days’ duration. Associated symptoms included fatigue, swelling of the feet and ankles, and migratory lower extremity joint pain. She denied any recent travel, sick contacts, or illnesses. Her past medical history was significant for asthma and seasonal allergies. Medications were limited to a daily multivitamin and turmeric supplements, which she recently discontinued. She denied the use of tobacco, alcohol, or illicit drugs. Her family history was non-contributory.

**Figure 1:**
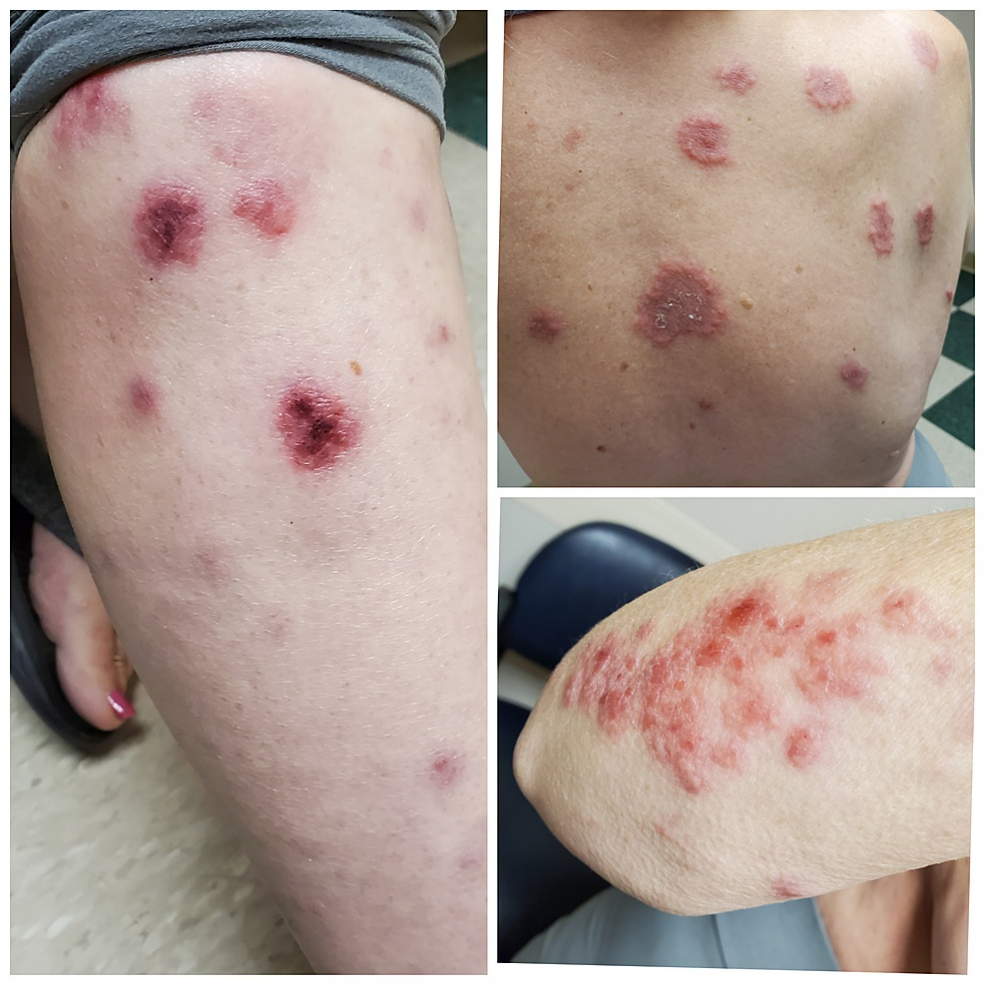
Erythematous, blanchable, palpable, annular papules and plaques on the back, right shin, and right arm

Skin examination revealed edematous, blanchable, palpable, red annular papules and plaques on the back and extremities, with the greatest amount present on the lower extremities (Figure 1). The lesions on the back had centripetal scaling. A resolving flesh-colored annular papule was present on the face. No active lesions were found in the mouth. There was 2+ pitting edema in the lower legs.

Her laboratory blood work was significant for hypoalbuminemia, an elevated erythrocyte sedimentation rate (ESR) of 70 mm/hour, and a positive ASO titer. The rapid strep test was negative. Chest x-ray was negative for any infiltrates, effusions, or cardiopulmonary abnormalities. Urinalysis was cloudy in appearance and positive for protein, ketones, and blood, and urine culture was sterile. Tests for antinuclear antibody, DNA double-stranded antibody, rheumatoid factor, HLA-B27, cyclic citrullinated peptide, Lyme, Babesia, Ehrlichia, Anaplasma, anti-Ro/SSA, anti-La/SSB, proteinase-3 anti-neutrophil cytoplasmic antibodies (ANCA), and myeloperoxidase ANCA were all negative. ESR returned to normal when rechecked four days later.

**Figure 2:**
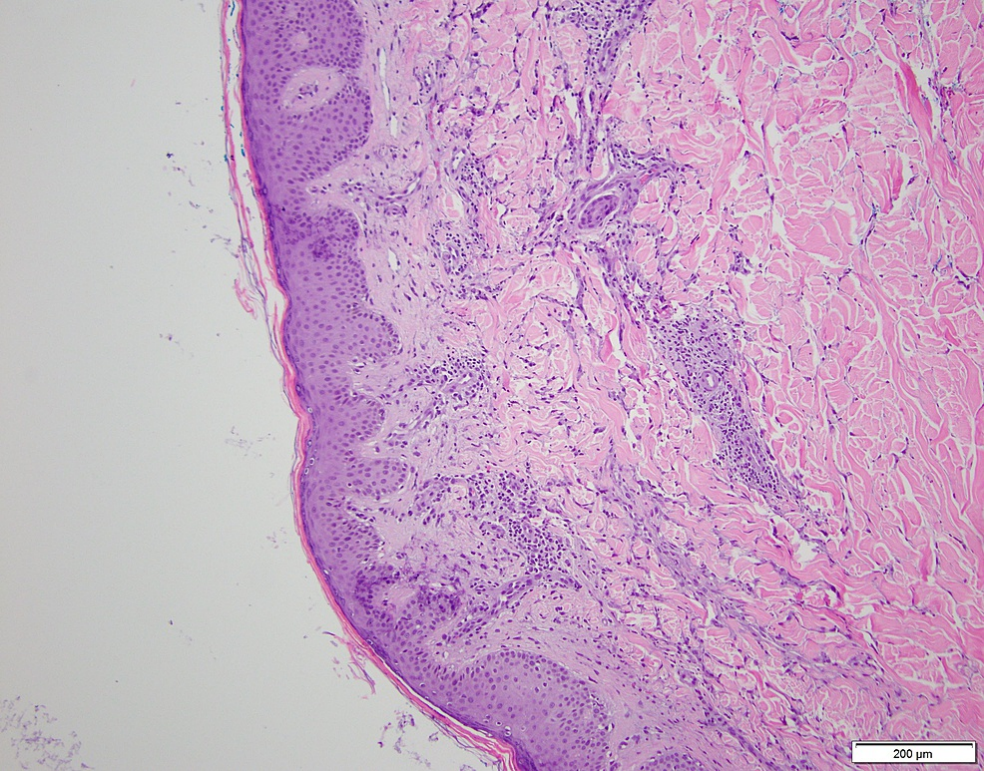
Low-power examination of a 4-mm punch biopsy from the patient's back shows a superficial perivascular inflammatory infiltrate composed of lymphocytes, neutrophils, and occasional eosinophils with abundant karyorrhectic debris and extravasated erythrocytes

A 4-mm punch biopsy was obtained from the patient's back. Pathology showed a superficial perivascular inflammatory infiltrate composed of lymphocytes, neutrophils, and occasional eosinophils (Figure 2). Abundant karyorrhectic debris was also seen with extravasated erythrocytes. While there was no evidence of active vascular destruction or fibrin deposition, the findings were suspicious for small-vessel vasculitis. Immunofluorescence was not performed.

Given the patient’s lower limb predominant papules, arthralgias, renal involvement, negative ANCA, and positive ASO titer, a diagnosis of IgA vasculitis was made. Treatment was initiated with an oral 40 mg prednisone taper over 21 days.

Two weeks later, the patient reported resolution of her fatigue, joint pain, and swelling. No new skin eruptions had occurred since beginning treatment and the previous lesions were faintly visible. Additional labs were ordered at her one-month follow-up, consisting of IgG, IgA, IgM, C3, C4, total serum protein with albumin, urine microalbumin, and urine creatinine, which were all within normal limits. At this point, she had completed her treatment regimen and all symptoms had resolved.

## Discussion

Adult onset IgA vasculitis differs in clinical presentation and treatment from that of the pediatric population. The classic triad of IgA vasculitis in children is palpable purpura, abdominal pain, and arthritis. In our case, the patient did not have abdominal pain, but rather had renal involvement as evident by the presence of proteinuria and hematuria. Upon further evaluation, the patient’s urine microalbumin and creatinine levels were normal, which was important to assess given the high prevalence of progression to end-stage renal disease in adults with IgA vasculitis. While the most likely diagnosis is IgA vasculitis, given the patient’s clinical picture, small-vessel vasculitis cannot be completely ruled out, as direct immunofluorescence staining was not performed.

The histological feature of IgA vasculitis is that of leukocytoclastic vasculitis, with a neutrophil predominant infiltrate with extravasated erythrocytes and fibrinoid necrosis. In addition, under direct immunofluorescence, IgA deposition in the walls of dermal blood vessels is found. While our biopsy did not necessarily demonstrate these findings, we suspect that this is due to the duration for which the papule was present. Biopsy should ideally be performed within 48 hours of a lesion developing for the best chance of displaying the histological findings [12]. In our case, the papule was present for more than 48 hours, thus showing a nonspecific lymphocyte predominant inflammatory reaction.

While treatment is often not warranted for IgA vasculitis, in this case, the patient was given systemic corticosteroids because of how widespread and distressing her lesions were. After two weeks of treatment, the patient had complete resolution of her fatigue, joint pain, and swelling, with no new lesions forming.

## Conclusions

This case demonstrates the importance of considering IgA vasculitis as a differential diagnosis in adults presenting with small-vessel vasculitis. While children often present with the classic triad of palpable purpura, abdominal pain, and arthritis, adults are more likely to have renal involvement, as was the case in this patient. Although corticosteroids can alleviate skin, joint, and abdominal symptoms, it is imperative to monitor renal function as current treatments are not effective at improving long-term renal outcomes.
